# A Prospective Study of Romanian Agriculture Workers for Zoonotic Influenza Infections

**DOI:** 10.1371/journal.pone.0098248

**Published:** 2014-05-28

**Authors:** Alexandru Coman, Daniel N. Maftei, Whitney S. Krueger, Gary L. Heil, Razvan M. Chereches, Emanuela Sirlincan, Paul Bria, Claudiu Dragnea, Iosif Kasler, Marissa A. Valentine, Gregory C. Gray

**Affiliations:** 1 Center for Health Policy and Public Health, Institute for Social Research, Faculty of Political, Administrative and Communication Sciences, Babes-Bolyai University, Cluj-Napoca, Romania; 2 College of Public Health and Health Professions, and Emerging Pathogens Institute, University of Florida, Gainesville, Florida, United States of America; Duke-NUS Gradute Medical School, Singapore

## Abstract

**Background:**

In this prospective study we sought to examine seroepidemiological evidence for acute zoonotic influenza virus infection among Romanian agricultural workers.

**Methods:**

Sera were drawn upon enrollment (2009) and again at 12 and 24 months from 312 adult agriculture workers and 51 age-group matched controls. Participants were contacted monthly for 24 months and queried regarding episodes of acute influenza-like illnesses (ILI). Cohort members meeting ILI criteria permitted respiratory swab collections as well as acute and convalescent serum collection. Serologic assays were performed against 9 avian, 3 swine, and 3 human influenza viruses.

**Results:**

During the two-year follow-up, a total of 23 ILI events were reported. Two subjects' specimens were identified as influenza A by rRT-PCR. During the follow-up period, three individuals experienced elevated microneutralization antibody titers ≥1∶80 against three (one each) avian influenza viruses: A/Teal/Hong Kong/w312/97(H6N1), A/Hong Kong/1073/1999(H9N2), or A/Duck/Alberta/60/1976(H12N5). However, none of these participants met the criteria for poultry exposure. A number of subjects demonstrated four-fold increases over time in hemagglutination inhibition (HI) assay titers for at least one of the three swine influenza viruses (SIVs); however, it seems likely that two of these three responses were due to cross-reacting antibody against human influenza. Only elevated antibody titers against A/Swine/Flanders/1/1998(H3N2) lacked evidence for such confounding. In examining risk factors for elevated antibody against this SIV with multiple logistic regression, swine exposure (adjusted OR = 1.8, 95% CI 1.1–2.8) and tobacco use (adjusted OR = 1.8; 95% CI 1.1–2.9) were important predictors.

**Conclusions:**

While Romania has recently experienced multiple incursions of highly pathogenic avian influenza among domestic poultry, this cohort of Romanian agriculture workers had sparse evidence of avian influenza virus infections. In contrast, there was evidence, especially among the swine exposed participants, of infections with human and one swine H3N2 influenza virus.

## Introduction

Since 2003, the World Health Organization has collected data from more than 600 human infections of highly pathogenic avian influenza (HPAI) H5N1 virus occurring in at least 15 countries. Case fatality rates as high as 60% have been reported, keeping public health warning systems on alert. Thus, when migrating birds, intermingling in Romania's Danube Delta introduced HPAI H5N1 into Romanian poultry in 2005, we initiated serology studies to screen Romanian agriculture workers and controls for avian influenza infection [1]. Since 45% of Romanians reside in rural areas, populated with small subsidence farms, a large population of Romanians may have experienced exposure to avian influenza viruses (AIVs) that may have spilled over from migrating birds into domestic birds [1]. A previous study examined serologic evidence of zoonotic influenza infection upon enrollment and found evidence of previous infections with avian-like A/Hong Kong/1073/1999(H9N2) [1]. This report presents data from 2 years of following the cohort.

## Materials and Methods

Details regarding the study location, study subjects, subject enrollment, database generation, and serology laboratory methods were previously published [1]. Briefly, the study targeted adults ≥18 years of age with intense and prolonged animal exposure from two regions in Romania: Tulcea County, located in the Danube Delta where enrollees were mostly associated with large, commercial swine confined animal feeding operations (CAFOs) (n = 149) and in Cluj County, where enrollees experienced animal contact mostly through traditional, small backyard farms (n = 163). The control population (n = 51) consisted of an age-group matched cohort recruited from Babes-Bolyai University in Cluj-Napoca, who denied having swine or poultry exposures. Those meeting animal exposure criteria and subjects in the control groups were matched based on the following age groups: 20–39, 40–59, and ≥60 years-old. After engaging local business or village leaders, study staff met with potential study subjects and explained the study and invited them to participate via informed consent. Upon enrollment, study staff collected sera and used a questionnaire to obtain demographic data, information about their medical history, community, household characteristics, and details regarding occupational and domestic animal exposure.

The cohorts established between February 2009 and January 2010 were followed monthly by telephone or face-to-face encounters over a 24-month period for evidence of influenza-like-illness (ILI). Sera and questionnaire data were collected at enrollment, 12 months, and 24 months. Annual follow-up questionnaires captured demographic, health, and animal exposure data during the previous year. Animal exposure was classified as close contact within one meter of domestic poultry, wild birds, or pigs as part of daily activities for ≥5 cumulative hours per week.

### Ethics statement

A total of four institutional review boards reviewed and approved the study: Babes-Bolyai University, University of Iowa, University of Florida, and Human Research Protection Office of the US Army Medical Research and Materiel Command. Written informed consent was obtained for each study participant.

### Monthly follow-up

Upon enrollment, participants were given oral and written instructions and a digital thermometer to help identify signs and symptoms of an ILI. ILI was defined as an acute onset of a respiratory illness with an oral (or equivalent from other body region) measured temperature ≥100.5°F (38°C) and a sore throat or cough, lasting for ≥4 hours. Participants experiencing symptoms were instructed to inform study staff upon development of an ILI. Additionally, study staff contacted the subjects each month to determine if any had experienced an ILI event.

### Investigating influenza-like illness

When a possible ILI was reported to study staff, a home visit was performed within 24 hours of notification. If the subject met the ILI case definition, a study nurse completed an ILI questionnaire and collected two respiratory swab specimens (nasal and pharyngeal) as well as acute and 2-month convalescent serum samples. The swab specimens were stored in viral transport media (Copan Diagnostics Inc., Murrieta, CA) and transported using cold-chain within 24 hours after collection to local field laboratories in either Tulcea or to Babes-Bolyai University.

### Laboratory methods

Sera and ILI respiratory swab aliquots were preserved at −80°C and transported on dry ice to the University of Florida for testing. Sera were tested for evidence of human, swine, and avian influenza infections over time using the best geographical and temporally-associated viruses we could access (Table 1). ILI swabs were studied for molecular evidence of influenza A. Influenza viruses, viral antigens, and control antisera were obtained from acknowledged collaborators, Biodefense and Emerging Infections (BEI) Research Resources Repository, or through the Influenza Reagent Resource (IRR) program of the US CDC.

**Table 1:** Viruses used in serological studies.

Avian viruses	Human viruses
A/Migratory duck/Hong Kong MPS180/2003(H4N6)	A/Brisbane/59/2007(H1N1)*
A/Nopi/Minnesota/2007/462960-2(H5N2)	A/Mexico/4108/2009(H1N1)* †
A/Teal/Hong Kong/w312/97(H6N1)	A/Brisbane/10/2007(H3N2)*
A/Water fowl/Hong Kong/Mpb127/2005(H7N7)	
A/Migratory duck/Hong Kong/MP2553/2004(H8N4)	Swine virus
A/Hong Kong/1073/1999(H9N2)**	A/Swine/Lutol/3/2000(H1N1)*
A/Migratory duck/Hong Kong/MPD268/2007(H10N4)	A/Swine/Gent/7625/1999(H1N2)*
A/Chicken/New Jersey/15906-9/1996(H11N1)	A/Swine/Flanders/1/1998(H3N2)*
A/Duck/Alberta/60/1976(H12N5)	

Unless otherwise indicated, serologic study was performed using the microneutralization assay.

*Virus studied with the hemagglutination inhibition assay.

† Similar to 2009 pandemic H1N1 virus.

**Virus of avian origin but cultured from a man.

#### Real-time RT-PCR influenza assay

Viral RNA was isolated from 140 µl of each swab specimen and processed using the Qiagen: QIAamp Viral RNA Mini Kit (Qiagen Inc., Valencia, California) mini-spin protocol, according to manufacturer's instructions. RNA was eluted in 50 µl of elution buffer. Specimens were screened for the presence of influenza A viral RNA using a SuperScript III One-Step RT-PCR System with Platinum Taq DNA Polymerase (Life Technologies, Grand Island, New York) with the Centers for Disease Control and Prevention's (CDC's) Human Influenza Virus Real-Time RT-PCR Detection and Characterization Panel [2]. The primer and dual labeled hydrolysis probes in this system are capable of universal detection of influenza A virus while subtyping primer and probe sets are designed to specifically detect contemporary human A/H1, human 2009 pandemic H1N1, human A/H3, and avian A/H5 (Asian lineage) influenza viruses. Each extraction run included a mock extraction control to provide a secondary negative control to validate the extraction procedure and reagent integrity. The human RNase P gene primer set was used as an internal positive control for human RNA in each sample. Specimens that were rRT-PCR positive for generic influenza type A were further evaluated with a rRT-PCR procedure specific for human H1, H3, and H5, as well as 2009 pandemic H1. Swab samples positive for influenza A, but unable to be subtyped, were cultured in Madin-Darby canine kidney (MDCK) cells and passaged twice in an attempt to amplify the virus for further study.

#### Hemagglutination inhibition (HI) assay

We employed the WHO-recommended HI assay [3] to test for serum antibodies against human and swine influenza A viruses. Influenza virus strains were grown in MDCK cells or fertilized eggs. Sera were pre-treated with receptor destroying enzyme (Denka Seiken Co., LTD., Tokyo, Japan) and hemabsorbed with either guinea pig or turkey erythrocytes. For seasonal human influenza virus strains, guinea pig erythrocytes were used in assay plates with a round bottom, or “U” shaped wells. For swine influenza viruses, turkey erythrocytes were used in plates with a conical bottom, or “V” shaped wells. Titer results were reported as the reciprocal of the highest dilution of serum that inhibited virus-induced hemagglutination of a 0.65% (guinea pig) or 0.50% (turkey) solution of erythrocytes [4].

#### Virus microneutralization (MN) assay

A WHO-recommended MN assay adapted from that reported by Rowe [5], [6] was used to detect human antibodies against avian viruses. Viruses at 100TCID50 as determined by the method of Reed and Muench [7] grown in embryonated chicken eggs, were combined with sera and 2×104 MDCK cells added to each neutralization reaction. Serum specimens were first screened at a dilution of 1∶10 and positive specimens were titrated in duplicate. After 24 hour incubation at 37°C plates were washed twice with PBS, fixed with cold 80% acetone, and allowed to dry at room temperature for 10 minutes. Un-neutralized influenza on the fixed monolayers was then quantified by an influenza A nucleoprotein-specific indirect ELISA. The neutralization endpoint ELISA was expressed as the reciprocal of the highest dilution of serum with optical density (OD) less than X, where X =  [(average OD of virus control wells) + (average OD of cell control wells)]/2. Test cells with an OD>2 times the cell control OD mean were considered positive for virus growth.

### Statistical methods

Having evaluated the prevalence of antibody against various influenza A viruses in the enrollment report [1], we move to a prospective focus upon acute influenza A infections in this work. Acute influenza infection was defined as either a) isolation of influenza virus from a respiratory specimen obtained when a patient had an ILI, b) rRT-PCR evidence of influenza from such specimens, or c) a fourfold or greater rise in antibody titer against influenza viruses as measured over time through examining serial annual sera and paired ILI sera, with a ≥1∶40 threshold for SIVs and human influenza viruses and a new threshold antibody titer of ≥1∶80 for AIVs. As done previously [8], [9], [10], [11], a HI titer ≥1∶40 was accepted as evidence of human or SIV infection or human influenza vaccination.

When analyzing serial sera for evidence of SIV, we used bivariate analysis to determine if any confounding existed between human influenza virus covariates and any of the SIVs. After identifying SIVs not markedly confounded by human influenza virus covariates, multivariate logistic regression was performed to determine risk factors associated within the individuals with evidence of infection with a SIV. When data were sparse, for any of the viruses examined, we employed an exact method. Data analysis was performed using SAS v9.3 (SAS Institute, Inc., Cary, NC, USA).

## Results

### Study population

Between February 2009 and January 2010, field staff collected demographic and serology data from 363 participants: 149 modern swine workers from Tulcea, 163 small farm workers from Cluj-Napoca and 51 age-group matched controls in this prospective study of zoonotic influenza infections [1]. See enrollment report for complete demographic characteristics of study participants. A total of 341 participants (94%) completed the 12-month annual follow-up and 286 participants (78.8%) completed their 24-month annual follow-up visit.

### Acute human influenza A infections

A total of 23 ILI investigations were conducted among 19 participants over the two-year period (5 participants had >1 ILI events). Among the 23 ILI events, two subjects' swabs were positive by rRT-PCR for human H3N2, while one of the two was also positive for pandemic influenza A H1N1. For the 23 ILI events, 10 paired acute and convalescent serum samples were collected and tested, but these ILI sera failed to reveal evidence of infection with a specific avian, swine, or human influenza virus.

### Serologic assay results for human influenza viruses

Upon examining serial annual sera for ≥4-fold increases in HI antibody titers against human influenza viruses, 86 (26%) had increases against A/Brisbane/59/2007(H1N1), 54 (16%) against A/Brisbane/10/2007(H3N2), and 144 (43%) against A/Mexico/4108/2009(pH1N1). Table 2 lists the serological reactivity against the three human influenza viruses for the overall population as well as comparisons between agriculture workers and non-agriculture workers. When comparing the serological reactivity between agriculture workers and non-agriculture workers, the odds of having elevated activity to any human influenza virus did not differ significantly between the occupational groups (Table 2).

**Table 2:** Serological activity against swine and human influenza viruses by the hemagglutination inhibition assay, 2009–2011.

Virus Strain	Total	Control	Agriculture Workers	Unadjusted OR
	N (%)	N (%)	N (%)	OR (95% CL)
A/Brisbane/59/2007(H1N1)*				
Positive	86 (26.1)	62 (25.7)	24 (27.3)	1.1 (0.6–1.9)
Negative	243 (73.9)	179 (74.3)	64 (72.7)	
A/Brisbane/10/2007(H3N2)*				
Positive	54 (16.3)	39 (16)	15 (17)	1.1 (0.6–2.1)
Negative	278 (83.7)	205 (84)	73 (83)	
A/Mexico/4108/2009(H1N1)*				
Positive	144 (43.4)	100 (41)	44 (50)	1.4 (0.9–2.4)
Negative	188 (56.6)	144 (59)	44 (50)	
A/Sw/Lutol/3/00(H1N1)*				
Positive	22 (6.9)	17 (7.4)	5 (5.7)	0.8 (0.3–2.1)
Negative	297 (93.1)	214 (92.6)	83 (94.3)	
A/Sw/Gent/7625/99(H1N2)*				
Positive	221 (65.5)	158 (64.8)	63 (72)	1.4 (0.8–2.3)
Negative	111 (34.5)	86 (35.2)	25 (28)	
A/Swine/Flanders/1/1998(H3N2)				
Positive	109 (32)	74 (29.2)	35 (39.8)	1.6 (1.0–2.7)
Negative	232 (68)	179 (70.8)	53 (60.2)	

Unadjusted odds ratio for agriculture workers against control enrollees with binary logistic regression.

*Covariate has some missing values.

### Serologic assay results for avian influenza viruses (AIVs)

Three subjects during the two-year follow-up period demonstrated a 4-fold MN titer rise against one of each of the following AIVs: A/Hong Kong/1073/1999(H9N2), A/Teal/Hong Kong/w312/97(H6N1), and A/Duck/Alberta/60/1976(H12N5) (Table 3). The individual with a 4-fold titer increase against A/Duck/Alberta/60/1976(H12N5) had titers of 1∶160 at both the 12- and 24-month follow-up periods. However, none of the participants with positive titers against AIV met the criteria for poultry exposure.

**Table 3:** Study participants with ≥4-fold increases in microneutralization titers against avian influenza viruses at enrollment, 12-month and 24-month follow-up, and associated risk factors.

Subject ID	0 Months	12 Months	24 Months	Poultry exposure	Home poultry exposure	Risk factors
H6N1						
521	<1∶10	1∶80	LTFU*	N	Y	RI†
H9N2						
638	<1∶10	1∶80	<1∶10	N	N	N
H12N5						
RJ625	<1∶10	1∶160	1∶160	N	N	N

H6N1 = A/Teal/Hong Kong/w312/97(H6N1); H9N2 = A/Hong Kong/1073/1999(H9N2).

H12N5 = A/Duck/Alberta/60/1976(H12N5).

*Lost to follow-up.

† Respiratory illness (fever and cough or sore throat) in previous 12 months.

### Serologic assay results for SIV

This study also investigated the seroreactivity of study participants' sera using the HI assay against three SIV strains: A/Swine/Lutol/3/2000(H1N1), A/Swine/Gent/7625/1999(H1N2), and A/Swine/Flanders/1/1998(H3N2). In terms of seroreactivity to SIVs during the two-year follow-up period, a number of subjects had ≥4-fold increases in antibody titers: 22 (7%) against A/Swine/Lutol/3/2000(H1N1), 109 (33%) against A/Swine/Flanders/1/1998(H3N2), and 221 (67%) against A/Swine/Gent/7625/1999(H1N2) (Table 2). When comparing serological reactivity against any SIV between agriculture workers and non-agriculture workers, the odds of having a ≥4-fold rise in antibody titer did not differ significantly between groups (Table 2).

Due to the large of amount of seroreactivity to SIVs in the cohort, we performed bivariate studies of association between each swine virus during the two-year follow-up against the following human influenza strains: A/Brisbane/59/2007(H1N1), A/Mexico/4108/2009(H1N1) and A/Brisbane/10/2007(H3N2). Both A/Swine/Gent/7625/1999(H1N2) and A/Swine/Lutol/3/2000(H1N1) positives during the two-year follow-up period were statistically associated with elevated antibodies against both A/Brisbane/59/2007(H1N1) and A/Brisbane/10/2007(H3N2), suggesting confounding from antibodies against human virus (Table 4). However, bivariate analysis of A/Swine/Flanders/1/1998(H3N2) against the three human influenzas revealed no significant association (Table 4). We then performed logistic regression analysis and found that tobacco product use (OR = 1.7, 95% CI: 1.1–2.8), and swine exposure (OR = 1.7, CI: 1.1–2.7) were independently associated with elevated antibodies against A/Swine/Flanders/1/1998(H3N2) (Table 5). Other risk factors examined, but found not to be statistically important included lack of indoor water, chronic respiratory illness, gender, and influenza vaccination during the past 12 months.

**Table 4:** Results from bivariate analyses performed between each SIV by each human influenza virus studied.

	A/Brisbane/59/2007(H1N1)	A/Brisbane/10/2007(H3N2)	A/Mexico/4108/2009(H1N1)
A/Sw/Lutol/3/00(H1N1)			
Chi-square p-value	0.001	0.012	0.358
OR (95% CI)	4.4 (1.8–10.8)	3.1 (1.2–7.9)	1.5 (0.6–3.6)
A/Sw/Gent/7625/99(H1N2)			
Chi-square p-value	0.006	<0.001	0.331
OR (95% CI)	2.2 (1.2–4)	6 (2.3–15.6)	1.3 (0.8–2)
A/Sw/Flanders/1/98(H3N2)			
Chi-square p-value	0.587	0.472	0.521
OR (95% CI)	1.2 (0.7–1.9)	1.3 (0.7–2.3)	1.2 (0.7–1.8)

**Table 5:** Risk factors for elevated antibodies against A/Swine/Flanders/1/1998(H3N2) among study participants from 2009–2011.

Variables	Total N	N (%)	Unadjusted OR (95% CI)*	Adjusted OR (95% CI)†
Indoor water**				
No	32	29.6	1.3 (0.8–2.3)	-----
Yes	76	70.3		
Tobacco products use				
Yes	39	35.8	1.7 (1.1–2.8)	1.8 (1.1–2.9)
No	70	64.2		
Swine Exposure				
Yes	66	60.6	1.7 (1.1–2.7)	1.8 (1.1–2.8)
No	43	39.4		
A/Brisbane/59/2007(H1N1)**				
Positive	30	28	1.2 (0.7–1.9)	------
Negative	77	72		
A/Brisbane/10/2007(H3N2)				
Positive	20	18.3	1.3 (0.7–2.3)	------
Negative	89	81.7		
A/Mexico/4108/2009(H1N1)				
Positive	50	45.9	1.2 (0.7–1.8)	------
Negative	59	54.1		

*Binary logistic regression (Negative =  H3N2 titer <1∶40, positive titer  = 4-fold increase ≥1∶40).

† Unconditional logistic regression.

**Covariate has missing data.

## Discussion

Romania, having recently joined the European Union, is quickly developing its poultry and pork industries to help support European demand for protein. This rapid growth has in some regions involved the development of modern confinement facilities for chicken and poultry as supported by large agribusinesses. This rapid growth has not been without biosecurity lapses. Romania has experienced poultry outbreaks of HPAI H5N1 in 2005, 2006, 2007, and 2010 [12], [13], [14] and a series of classical swine fever outbreaks from 2005 to 2007 among pigs [15]. Realizing that surveillance for AIV and SIV is sparse in Romania and biosecurity in the poultry and swine industries as well as in small farms may be developing, this study was designed to examine evidence of AIV or SIV infections among Romanian agriculture workers.

Unlike the cross-sectional seroepidemiological study of this cohort where some evidence of H9N2 infections in cohort members was found [1], relatively little evidence of AIV infection was found after enrollment. Only 3 individuals were found to have new, elevated MN titers against 3 AIVs (one each) over the 2-year period. While one of the three subjects reported both having an illness during the follow-up period and household exposure to poultry, the participant did not meet the criteria for poultry exposure (≥5 hours per week). It is unclear if these rises in MN titers represented true AIV infection or a laboratory assay anomaly.

Regarding SIVs, annual sera did show strong serological reactivity to the three SIVs. However, cross-reactivity against human influenza viruses likely explained elevated antibody against two of the SIVs. Only elevated antibody against A/Swine/Flanders/1/1998(H3N2) seemed not to be strongly confounded by antibody against human influenza viruses. Multivariate analyses suggested that swine-exposed participants and tobacco users were at increased risk. In previous studies among US pig workers [10], we have similarly found elevated antibodies against SIV which we have hypothesized may be due to autoinoculation of the workers' oral mucosa when lighting a cigarette after touching pig secretions.

This study has a number of limitations. Using serological studies to determine evidence of AIV or SIV infection can pose a problem in that elevated titers may actually reflect cross-reacting antibodies from human influenza viruses or vaccines. We attempted to control for this by screening through bivariate analyses for such confounding, but our adjustments were likely imperfect as they were limited to examining only 3 human viruses. The study is further limited in that the viruses used to examine serological reactivity could have differed from viruses circulating amongst the Romanian study participants, as Romania does not perform active surveillance for influenza A viruses among poultry or pigs. Additionally, as in the enrollment paper, due to the nature of this occupational investigation, only adults 18 years or older were included in the study. Children are often more susceptible to influenza infection due to less developed immune systems. Hence, we may have missed risk factors by basing our study on adults. Finally, the low serological reactivity towards AIV could have been due to the low sensitivity of the MN assay against AIVs.

## Conclusions

For 2 years we prospectively followed a controlled cohort of Romania's agriculture workers from two quite different geographical areas and found only a few study participants with evidence of acute AIV infection. We also studied the participants for evidence of incident infections with 3 swine viruses and found some evidence of human infection with A/Swine/Flanders/1/1998(H3N2), but again it was not as compelling as studies we have performed in the USA [16], [17]. In short, evidence of human AIV and SIV infections in this Romanian cohort was relatively sparse.
